# Increasing Dosage of Leucovorin Results in Pharmacokinetic and Gene Expression Differences When Administered as Two-Hour Infusion or Bolus Injection to Patients with Colon Cancer

**DOI:** 10.3390/cancers15010258

**Published:** 2022-12-30

**Authors:** Helena Taflin, Elisabeth Odin, Göran Carlsson, Bengt Gustavsson, Yvonne Wettergren, Elinor Bexe Lindskog

**Affiliations:** 1 Department of Surgery, Institute of Clinical Sciences, Sahlgrenska University Hospital, Sahlgrenska Academy at University of Gothenburg, 41685 Gothenburg, Sweden; 2 Transplant Center, Sahlgrenska University Hospital, 41345 Gothenburg, Sweden

**Keywords:** pharmacokinetics, leucovorin, two-hour infusion, bolus injection, colon cancer, gene expression, LC-MS/MS, methylenetetrahydrofolate

## Abstract

**Simple Summary:**

5-fluorouracil (5-FU) is the drug used in almost all chemotherapy regimens for colorectal cancer. To potentiate the drug effect, 5-FU is most often combined with the folate leucovorin (LV). Although 5-FU dosage to some extent is individualized, LV dosage is not. To address the issues of LV dosage and administration this randomized pharmacokinetic study was performed comparing different LV dosages as well as infusion and bolus injection administration regimens. Folate concentrations in blood, tumor, mucosa and resection margins were analyzed as was tissue gene expression. In conclusion, different metabolic mechanisms appear to be induced when LV is administered as two-hour infusion or bolus injection, respectively. To achieve maximal inhibition of the 5-FU target enzyme thymidylate synthase (TS), an excess of the polyglutamated folate metabolite methylenetetrahydrofolate (MeTHF) is needed. Regarding polyglutamation, our results points in favor of the bolus regimen. These results could be used to optimize the effect of 5-FU.

**Abstract:**

The combination of 5-fluorouracil (5-FU) and leucovorin (LV) forms the chemotherapy backbone for patients with colorectal cancer. However, the LV administration is often standardized and not based on robust scientific data. To address these issues, a randomized pharmacokinetics study was performed in patients with colon cancer. Thirty patients were enrolled, receiving 60, 200 or 500 mg/m^2^ LV as a single two-hour infusion. Blood, tumor, mucosa, and resection margin biopsies were collected. Folate concentrations were analyzed with LC-MS/MS and gene expression with qPCR. Data from a previous study where patients received LV as bolus injections were used as comparison. Saturation of methylenetetrahydrofolate (MeTHF) and tetrahydrofolate (THF) levels was seen after two-hour infusion and polyglutamated MeTHF + THF levels in tumors decreased with increasing LV dosage. The decrease was associated with decreased *FPGS* and increased *GGH* expression, which was not observed after LV bolus injection. In the bolus group, results indicate activation of a metabolic switch possibly promoting TYMS inhibition in response to 5-FU. Different metabolic mechanisms appear to be induced when LV is administered as infusion and bolus injection. Since maximal inhibition of TYMS by the 5-FU metabolite 5-fluoro-2′-deoxyuridine 5′-monophosphate (FdUMP) requires excess polyglutamated MeTHF, the results point in favor of the bolus regimen.

## 1. Introduction

Colon cancer is one of the most common malignancies worldwide, and approximately 1.1 million people were diagnosed with the disease in 2018 [1]. Surgery remains the primary modality of treatment for malignancies of the lower gastrointestinal tract, and standard resection is today the only therapy required for early-stage cancer. As the tumor stage advances in terms of depth of penetration and lymph node involvement, the chance of cure with surgery alone diminishes and the rate of recurrence increase. 

Chemotherapy is used in all settings; neoadjuvant, with the intent of reducing the tumor burden and making the tumor resectable/less advanced, in the adjuvant setting after radical surgery to reduce the risk of recurrence, and in the pure palliative setting with the purpose of prolonging life [2,3]. Although novel combination treatments have been developed during the years, 5-FU in combination with the folate [6R,S]-5-formyltetrahydrofolate, i.e., leucovorin (LV), is still a cornerstone in treatment of colon cancer as well as several other gastrointestinal cancers [4].The racemic mixture of the natural (S) and unnatural (R) diastereoisomers of LV is most often used although the S-form is also commercially available. LV is metabolized intracellularly to the active metabolite 5,10-methylenetetrahydrofolate (MeTHF). 

**Figure 1:**
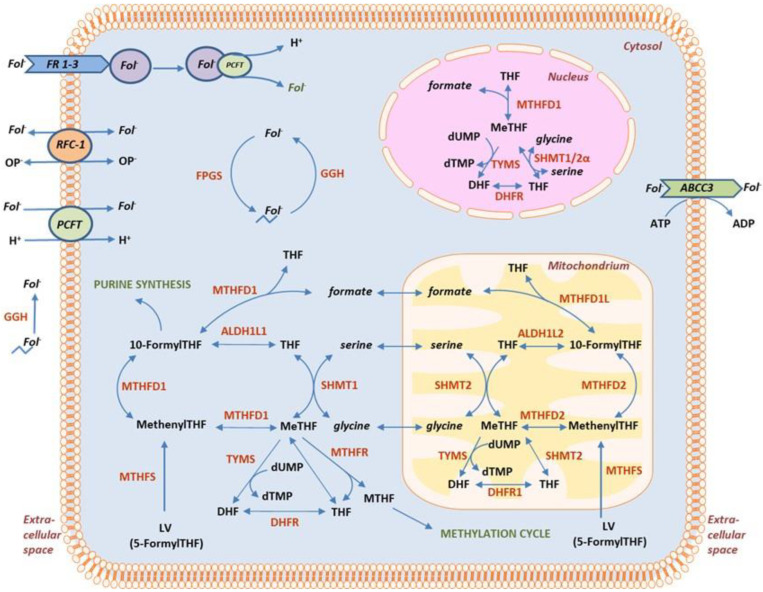
Simplified overview showing compartmentalization of folate metabolism in eukaryotic cells. Monoglutamated folates (*Fol^−^*), including leucovorin (LV), enter cells through RFC-1, PCFT, or FRs. Inside cells, polyglutamation of folates occur through the action of FPGS, whereas GGH yields monoglutamated folates. These may be exported out of cells by ABCC3. In the cytosol, LV may be enzymatically converted to MeTHF by two different routes: either by MTHFS and MTHFD1, or by MTHFS, MTHFD1, ALDH1L1, and SHMT1. Similar reactions occur in the mitochondria, where LV can be converted to MeTHF by MTHFS and MTHFD2, or by MTHFS, MTHFD2, ALDH1L2, and SHMT2. De novo dTMP synthesis in the cytosol occurs through the activities of the enzymes SHMT1, TYMS, and DHFR, and in mitochondria through SHMT2, TYMS, and DHFRL1 localized to the mitochondrial matrix and inner membrane. In the nucleus, sumoylated forms of SHMT1, TYMS, and DHFR forms a complex needed for nuclear folate metabolism. Abbreviations: *ABCC3*: ATP-binding cassette, subfamily C (CFTR/MRP), member 3; *ALDH1L1/2*: aldehyde dehydrogenase 1 family member L1/2; *DHF*: dihydrofolate; *DHFR*: dihydrofolate reductase; *dUMP*: deoxyuridine monophosphate; *dTMP*: deoxythymidine monophosphate; *FPGS*: folylpolyglutamate synthase; *Fol^−^*: monoglutamated folate; *FR*: folate receptor; *GGH*: gamma-glutamyl hydrolase; *MeTHF*: methylenetetrahydrofolate; *MTHFD1L*: methylenetetrahydrofolate dehydrogenase (NADP+ dependent) 1-like; *MTHFD2*:methylenetetrahydrofolate dehydrogenase (NAPD+ dependent) 2; *MTHFS*: 5,10-methenyltetrahydrofolate synthetase; *OP^−^*: organic phosphate; *PCFT*: solute carrier family 46 (folate transporter), member 1, proton coupled folate transporter; *RFC-1*: solute carrier family 19 (folate transporter) member 1; *SHMT1/2/2α*: serine hydroxymethyltransferase 1/2/2α; *TYMS*: thymidylate synthase; *THF*: tetrahydrofolate.

After entering the cells, 5-FU is converted to 5-fluoro-2′-deoxyuridine 5′-monophosphate (FdUMP), which forms an inhibitory ternary complex with MeTHF and the enzyme thymidylate synthase (TYMS). This results in the inhibition of thymidylate synthesis and impairment of both DNA synthesis and DNA repair. FdUMP-TS complex formation is dependent upon the cellular concentration of MeTHF. The combination of 5-FU and LV (FLV) leads to an increased intracellular pool of MeTHF and improved 5-FU cytotoxicity, as it shifts the equilibrium towards the formation of the ternary FdUMP-TS complex, ensuring maximum TS inhibition (Figure 1).

5-FU was in the beginning administered as a rapid bolus injection. Since the discovery that LV can modulate and increase the response rate in patients with metastatic colon cancer, the combination of 5-FU with LV has been standard and different administration regimens have been established [5]. These include 5-FU bolus injections for two-three minutes and infusion of LV for two hours. When infusion pumps became available, continuous infusion of 5-FU could proceed for up to 46 hours. Globally, a modified deGramont schedule starting with a two-hour infusion of LV and a rapid bolus injection of 5-FU, followed by a continuous infusion of 5-FU for 46 h, is frequently used as treatment for colon cancer [6]. The regimens have not been proven different regarding effectiveness, but a difference in adverse events has been seen [7]. Furthermore, the infusion pump-based regimens, which often require a central venous line, are more time consuming for both patients and the healthcare system.

The chosen LV dosages and/or the timing of LV administration used are not based on robust scientific data as the ability to measure the low intracellular concentrations of different reduced folate metabolites in blood and tissue has historically been limited. This has led several clinics to adopt flat-fixed dosing of LV. Determination of plasma and tissue folate concentrations using liquid chromatography/mass spectrometry (LC/MS) [8] or other sensitive techniques [9] may provide the opportunity to individualize folate dosing.

Still, there are few publications on the subject and a lack of knowledge about the effect of different LV dosages and administration regimens on expression of folate-associated genes. To address these issues, a randomized, exploratory study of the pharmacokinetics of 60, 200 or 500 mg/m^2^ racemic LV, given as a two-hour infusion before surgery, was conducted in patients with colon cancer. The effect of LV on folate levels in blood as well as in tumor and mucosa tissue was measured using LC-MS/MS and the results were related to expression of folate-associated genes in tissues. Data from a previous study, where LV was given preoperatively as a rapid bolus injection were used as comparison [10].

## 2. Materials and Methods

### 2.1. Patients

Thirty patients diagnosed with colon cancer and scheduled for elective colon resection during the years 2016–2020 were enrolled in the study. Patients were pre-operatively randomized into three groups: 60, 200 and 500 mg/m^2^ LV given as a single intravenous two-hour infusion starting preoperatively and continuing during surgery. The patients were otherwise treated in accordance with normal routines and guidelines. Blood samples for pharmacokinetic analysis were collected immediately prior to LV administration as well as 10, 30, 120, 240 minutes and 24 h after start of infusion. During surgery, at time of removal of the surgical specimen, biopsies from tumor and macroscopically normal-appearing mucosa 10 cm from the tumor, as well as from the resection margin, were collected and snap-frozen in liquid nitrogen and stored at −80 °C until analyzed. Clinical data were retrieved from patients’ medical records. Clinical and experimental data deriving from colon cancer patients (n = 38) in a previous study [10] were used for comparison. In that study, the patients were randomized to four groups receiving 0 (baseline), 60, 200 or 500 mg/m^2^ LV, given as bolus injections at the time of start of surgical procedure. A comparison of clinical and histopathological characteristics of the two study groups is presented in Table S1.

### 2.2. Materials

Potassium phosphate buffer, sodium ascorbate, ß-mercaptopropanol, aminoacetophenone, acetic acid (HAc), methanol, conjugase, acetonitrile, Tris(2-carboxyethyl)phosphine hydrochloride solution (TCEP), and dimethyl sulfoxide (DMSO) were purchased from Sigma Aldrich Sweden AB, Stockholm, Sweden. The folate standards 5-formylTHF (LV), 5,10-MeTHF, THF, and 5-MTHF were obtained from Merck Chemicals and Life Science AB, Solna, Sweden. TaqMan gene expression assays were purchased from ThermoFisher Scientific, Stockholm, Sweden. 

### 2.3. Plasma Folate Analyses

Blood samples were collected from 30 patients who received a two-hour infusion with 60, 200, or 500 mg/m^2^ LV at the following time points: t = 0, 10, 30, 120, 240 min, and 24 h. The samples were collected in EDTA vacutainers and immediately centrifuged at 2000g, 4 °C, for 10 min for collection of plasma. A blood sample was also collected from a healthy volunteer to be used for preparation of control plasma. The plasma samples were stored at −80°C until analysis. 

Samples were prepared by adding 10 µL distilled water, 10 µL aminoacetophenone (internal standard, IS) and 250 µL 10 mM TCEP diluted in methanol:DMSO (50:50) to 10 µL plasma. Control plasma was used to prepare low, medium, and high quality (Q) controls. Ten µL standard, 10 µL IS and 250 µL 10 mM TCEP diluted in methanol:DMSO (50:50) were added to 10 µL control plasma. The tubes with the solutions were mixed well for at least 5 min, followed by centrifugation at 3500× *g*, 4 °C, for 10 min. The supernatant was transferred to a new tube which was centrifuged at 21,500× *g*, 4 °C, for 10 min. The supernatant was then analyzed on the LC-MS/MS instrument. 

The LV and MTHF standards were serially diluted in extraction buffer (50 mM phosphate buffer, 1% sodium ascorbate, and 0.1% β-mercaptopropanol) to prepare the calibration curves. Intra-batch variability was determined by analyzing plasma Q-samples at low, medium, and high concentrations on the same day. Inter-assay variability was determined by analyzing low, medium, and high concentration samples on four separate days. The relative standard deviation (RSD) ranged from 2 to 5% for all analyses, and the variability over four days ranged from 6 to 11% for all analyses. The accuracy of the method was determined by estimating the recovery by adding known amounts of the standard to a sample. The average recovery was 87% for MTHF and 85% for LV.

### 2.4. Tissue Folate Analyses

On the day of sample analysis, an extraction buffer containing 50 mM phosphate buffer (pH 7.0), 1% ascorbate, and 0.1% β-mercaptopropanol was prepared. The tissue was weighed and placed in an Eppendorf tube and a 10× volume of extraction buffer was added. Homogenisation was performed using a TissueLyzer (two disruption steps at 25 Hz for 2.5 min each). Two-hundred µl of the homogenate were transferred to a new tube and mixed with 16 µl Tomudex which was used as IS. Next, the samples were incubated at 100 °C for 1 min followed by rapid cooling on ice. 

In order to analyze mono- and polyglutamated folates separately, each sample was split into two which were run in parallel. Twenty-five µl of conjugase were added to one of these samples, whereas 25 µL extraction buffer were added to the other. Each sample was then incubated at 37 °C for 60 min. After the deconjugation step, the samples were protein-precipitated, centrifuged, and ultrafiltrated at 21,500× *g*, 20 °C, for 30 min. The solution at the bottom of the tube was used for the LC-MS/MS analysis. 

The standards and samples were processed using the QuanLynx quantitative processing tool in MassLynx (Waters Corp., Milford, MA, USA). The levels of THF, MeTHF, MTHF and LV in each sample were expressed as pmol/g wet-weight (pmol/g_ww_). The relative standard deviation (RSD) ranged from 4 to 9% for all analyses, and the variability over four days ranged from 7 to 14%. The accuracy of the method was determined by estimating the recovery of known amounts of the standard added into a sample. The average recoveries were 105, 101, 83 and 86% for THF, MeTHF, MTHF and LV, respectively. 

### 2.5. LC-MS/MS Conditions

LC-MS/MS was used to determine the levels of the folate derivatives; THF, MeTHF, MTHF, and LV in tissue and plasma. The analyses were performed on a Waters 2795 LC separation module coupled to a Waters micromass Quattro Triple-Quadrupole MS system with an electrospray ionisation (ESI) source. Folates were detected and quantified using positive electrospray. The separation of folates was performed using an Atlantis dC_18_ 3 µm, 2.1 × 100 mm column (Waters) together with the guard column Atlantis dC_18_, 3 µm, 2.1 × 10 mm (Waters). A mobile phase consisting of eluent A (0.1% of acetic acid in water) and eluent B (0.1% acetic acid in acetonitrile) was used. The extracted ions following MRM transitions in plasma samples were monitored at 460 → 313 m/z for MTHF, 474 → 327 m/z for LV, and 136.03 → 94.1 m/z for para-aminoacetophenone (IS). The extracted ions following MRM transitions in tissue samples were monitored at 446 → 299 m/z for THF, 458 → 311 m/z for MeTHF, 460 → 313 m/z for MTHF, 474 → 327 m/z for LV, and 459 → 312 m/z for Tomudex (IS). 

### 2.6. RNA Extraction, cDNA Synthesis and Real-Time Quantitative PCR

Total RNA was isolated using Qiagen AllPrep DNA/RNA/protein mini kit (no. 80004, Qiagen GmbH, Hilden, Germany) according to the manufacturer’s instructions. cDNA was synthesized from total RNA using the High Capacity cDNA Reverse Transcription Kit (Applied Biosystems) and run on a Bio-Rad T100 Thermal Cycler (Bio-Rad laboratories, Solna, Sweden). The relative expression of the following selected genes involved in LV transport, metabolism and polyglutamation was quantified: ATP-binding cassette, subfamily C (CFTR/MRP), member 3 (*ABCC3*), solute carrier family 19 (folate transporter) member 1; reduced folate carrier 1 (*RFC-1*), solute carrier family 46 (folate transporter), member 1; proton coupled folate transporter (*PCFT*), methylenetetrahydrofolate dehydrogenase (NADP+ dependent) 1-like (*MTHFD1L*), methylenetetrahydrofolate dehydrogenase (NAPD+ dependent) 2 (*MTHFD2*), 5,10-methenyltetrahydrofolate (*MTHFS*), serine hydroxymethyltransferase 1 (*SHMT1*), thymidylate synthase (*TYMS*), folylpolyglutamate synthase (*FPGS*), gamma-glutamyl hydrolase (*GGH*), beta-actin (*ACTB*), and glyceraldehyde 3-phosphate dehydrogenase (*GAPDH*). The qPCR was set up as duplicates in 384-well plates using a Nanodrop II (GC Biotech) and was carried out in 5 µl reactions with 1×TaqMan^®^ Gene Expression Mastermix (Applied Biosystems), 1×gene-specific TaqMan assay (Applied Biosystems), and 1 µl cDNA. Assay IDs are presented in Table S2. The qPCR was run on a QuantStudio^TM^12K Flex Real-Time PCR System (Life Technologies) at TATAA Biocenter AB, Gothenburg, Sweden, according to a standard protocol. To compensate for between-run variations, values were adjusted to a control sample. The relative gene expression (ΔCt) was calculated by relating the mean Ct value of each target gene to a mean Ct value representing both housekeeping genes, to keep the variance to the minimum.

### 2.7. Statistics

The obtained data were analyzed by statistical modelling using the commercial software JMP 15.0.0 (SAS Institute, 2019) or Graph Pad Prism version 9.3.1, 2021. Data are presented as the median with a 95% confidence interval. Differences between groups were tested using the Wilcoxon Kruskal–Wallis test, the Mann–Whitney U test, the Pearson test, or the Wilcoxon matched-paired signed rank test. To compare sets of continuous parameters measured in the same sample, the Pearson correlation coefficient (r) was used. *p*-values < 0.05 were considered significant. The area under the plasma folate concentration-time curve (AUC) was calculated using the data obtained at different time points (0, 10, 30, 120, 240 min, and 24 h) using the pharmacokinetic software PKSolver (version 2).

## 3. Results

### 3.1. Demographic

The demographic of the patients is presented in Supplementary Table S1. In both the bolus and the infusions group, the patients were included in a consecutive order. No significant differences were seen regarding age, gender or stage of the diesase. There was a significant difference in sidedness, with more patients with right-sided tumors in the bolus group, compered to the infusion group (*p* = 0.016).

### 3.2. Plasma Folate Levels after Two-Hour Infusion of LV

**Figure 2:**
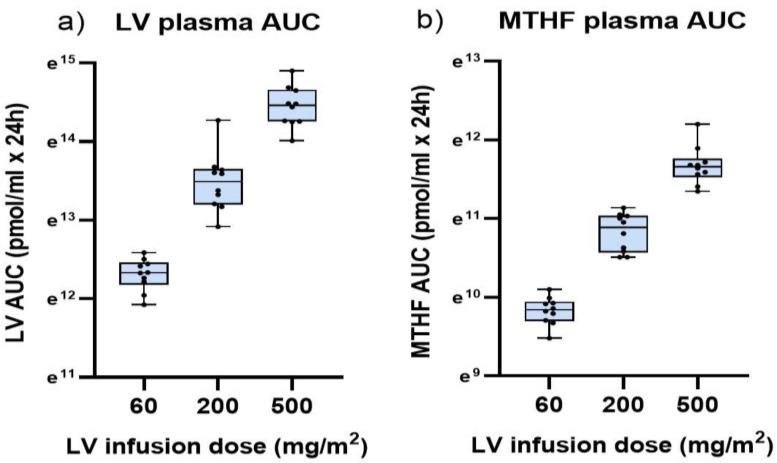
Dose-AUC relationship among patients receiving a two-hour intravenous infusion of 60, 200, or 500 mg/m^2^ LV. The figure (**a**) shows the AUC (pmol/mL × 24 h) of LV and the figure (**b**) shows the AUC of MTHF in plasma of the 30 included patients. Due to the large difference in LV and MTHF levels, the scale is based on the natural logarithm.

The LV plasma AUC levels (Figure 2a) and MTHF plasma AUC levels (Figure 2b) increased significantly with increasing two-hour infusion dose of LV (*p* < 0.0001). A strong correlation was seen between plasma LV AUC and MTHF AUC (r = 0.95, *p* < 0.0001). When subgrouping on dose, a correlation was seen between plasma LV AUC and MTHF in the group reciving 500 mg/m^2^, (r = 0.88, *p* = 0.0009), but not in the subgroups reciving 60 mg/m^2^ and 200 mg/m^2^ LV. 

### 3.3. Folate Levels in Tissue Samples after Two-Hour Infusion of LV

There was no significant difference in LV levels between tumor and mucosa of patients receiving 200 mg/m^2^ LV. Patients treated with 60 or 500 mg/m^2^ LV, however, had significantly higher LV concentrations in mucosa compared to tumor tissue (Table 1). The ratio between mucosa and tumor tissue LV concentrations increased with increasing dose of LV (1.64 (0.76–4.33) at 60, 1.74 (0.47–9.98) at 200, and 2.39 (0.65–4.98) at 500 mg/m^2^).

### 3.4. Folates in Plasma versus Tissue after Two-Hour Infusion of LV

The levels of MTHF, MeTHF, THF, and LV were measured in tumor and mucosa tissue of patients receiving two-hour infusion of LV and related to plasma folates. Since MeTHF is converted to THF over time, the sum of these two folate metabolites was calculated and is hereafter presented as (MeTHF + THF). The plasma LV and MTHF AUC values correlated with the MTHF levels (r = 0.67, *p* < 0.0001 and r = 0.71, *p* < 0.0001, respectively), but not the (MeTHF + THF) levels, in tumors. Upon subgrouping, a positive correlation was found between plasma LV AUC as well as MTHF AUC and MTHF in tumors of patients subjected to 500 mg/m^2^ LV (r = 0.71, *p* = 0.022 and r = 0.81, *p* = 0.0042, respectively). There was a significant correlation between plasma LV and MTHF AUC values and levels of MTHF (r = 0.60, *p* = 0.0004 and r = 0.61, *p* = 0.0003, respectively) in mucosa. Furthermore, plasma LV AUC as well as MTHF AUC correlated with the level of (MeTHF + THF) in mucosa (r = 0.51, *p* = 0.004, and r = 0.47, *p* = 0.0094, respectively). However, the correlation in mucosa was not seen when patients were grouped by LV dosage. 

**Figure 3:**
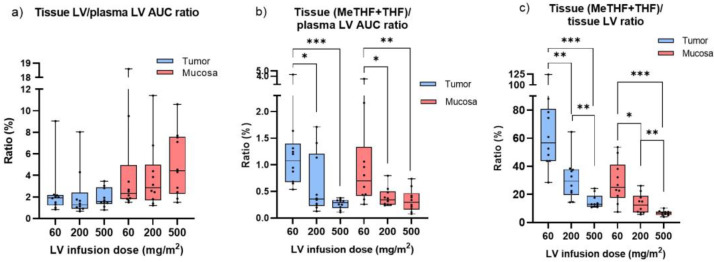
Relationship between (**a**) tissue LV level and plasma LV AUC, (**b**) tissue (MeTHF + THF) level and plasma LV AUC, and (**c**) tissue (MeTHF + THF) and LV levels, at different two-hour infusion dosages of LV. * *p* ≤ 0.05; ** *p* ≤ 0.01, *** *p* ≤ 0.001.

The “mucosa LV/plasma LV AUC” ratio was higher than the “tumor LV/plasma LV AUC” ratio (Figure 3a). It was also noted that there was no significant difference in the tissue LV/plasma LV AUC ratio according to LV dosage. In contrast, the “tissue (MeTHF + THF)/plasma LV AUC” ratio decreased significantly with increasing LV dosage in both tumor and mucosa (Figure 3b). This was also the case for the “tissue (MeTHF + THF)/plasma MTHF AUC” ratio. Furthermore, the “tissue (MeTHF + THF)/tissue LV” ratio decreased by dosage in both tumor and mucosa (Figure 3c).

### 3.5. Tissue Folate Levels at Baseline and after Two-Hour Infusion versus Bolus Injection

The MTHF and (MeTHF + THF) levels were compared in tumor and mucosa of patients at baseline. The median MTHF level was 124 (54–345) pmol/g in tumors and 224 (68–436) pmol/g in mucosa (*p* = 0.0098), whereas the level of (MeTHF + THF) was 1009 (407–1911) pmol/g in tumors and 867 (345–1251) pmol/g in mucosa (*p* = 0.13). The influence of two-hour infusion versus bolus injection of LV on folate concentrations in mucosa and tumor tissue are presented in Table 2. As shown, the MTHF level in patients receiving two-hour infusion was significantly higher in both tumor and mucosa compared to patients receiving bolus injections, at all LV dosages. However, no significant differences in (MeTHF + THF) levels were seen. No difference in MTHF levels was seen between tumor and mucosa, neither after two-hour infusion nor after bolus injection of LV. The levels of (MeTHF + THF) were significantly higher in tumors compared to mucosa after bolus injection, but not after two-hour infusion (Table 2).

### 3.6. Folate and Gene Expression Levels in Tissues at Baseline

**Figure 4:**
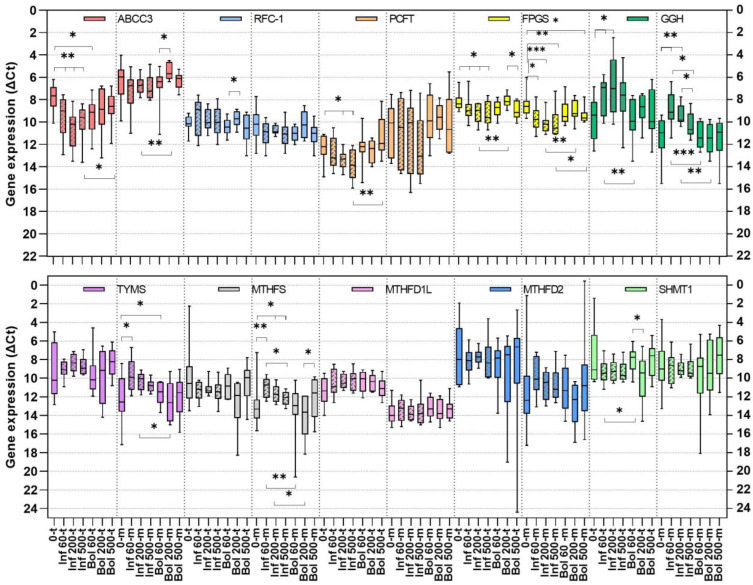
Box plots showing median gene expression levels in tumors (t) and mucosa (m) of colon cancer patients at baseline (0, n = 10) and after administration of 60 mg/m^2^ (Inf n = 10, Bol n = 10), 200 mg/m^2^ (Inf n = 10, Bol n = 8), or 500 mg/m^2^ (Inf n = 10, Bol n = 10) LV administered as two-hour infusion (Inf, shaded boxes) or bolus (Bol) injections. Significance asterisks above boxes show differences in tumors or mucosa related to LV dosage whereas significance asterisks below boxes show differences in tumors or mucosa relating to LV administration (two-hour infusion or bolus injection) at a specific LV dosage. * *p* ≤ 0.05; ** *p* ≤ 0.01, *** *p* ≤ 0.001.

The median expression level of each analyzed gene at baseline was compared in tumor and mucosa (Figure 4). Significantly higher levels of *TYMS* and *MTHFD1L* gene expression were found in tumors compared with mucosa (*p* = 0.020 and 0.014, respectively). The difference in expression of the other analyzed genes did not reach significance when tumor and mucosa tissue was compared.

### 3.7. Gene Expression Alterations in Tumor and Mucosa at Increasing Dosage of LV

To establish if the LV dosage influence folate levels by altering the expression of folate-associated genes, the gene expression levels were determined in tumor and mucosa of patients treated with increasing LV dosages and compared with baseline levels. The median *ABCC3* and *PCFT* expression was significantly lower in tumors compared to mucosa after two-hour infusion as well as bolus injection of LV (Figure 4). It was noted that the expression pattern of *ABBC3* and *PCFT* was synchronized after LV administration in both tumor and mucosa. With exception of *SHMT1*, all other genes had a higher expression in tumors compared to mucosa. Compared to baseline, *ABCC3* and *FPGS* expression in tumors was lower at all dosages after two-hour infusion whereas *PCFT* expression was lower at 200 and 500 mg/m^2^ and *GGH* expression higher at 60 and 200 mg/m^2^. No significant differences were found when baseline expression levels in tumors were compared with levels after bolus injection of LV. 

In mucosa, the major differences in gene expression were lower *FPGS* and higher *GGH* gene expression after two-hour infusion compared to bolus injection and baseline. Furthermore, the expression of *TYMS*, *MTHFS*, and *MTHFD2* was higher after two-hour infusion compared to baseline or bolus administration. It was also noted that the expression of *GGH*, *TYMS*, *MTHFS*, and *MTHFD2* in mucosa after two-hour infusion of LV followed the same pattern with the highest level at 60 mg/m^2^ and the lowest at 500 mg/m^2^. 

### 3.8. Correlation between Gene Expression and Folate Levels

**Figure 5:**
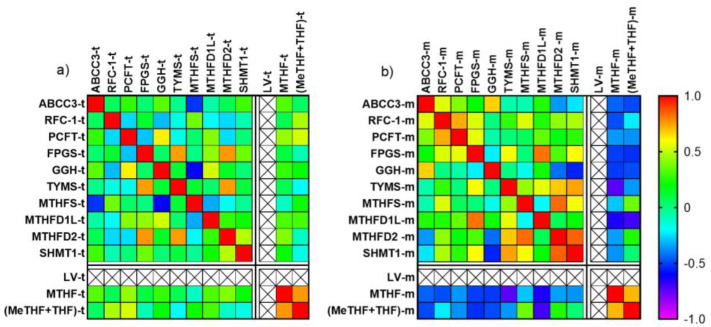
Heat maps showing the correlation between gene expression and folate levels in (**a**) tumors (t) and (**b**) mucosa (m) at baseline. The strongest positive correlation (1.0) is shown in red whereas the strongest negative correlation (−1.0) is shown in purple. LV in tissue was not analyzed at baseline.

The correlation between gene expression and folates levels in tumor and mucosa tissue at baseline is presented as heat maps in Figure 5 (correlation coefficients and *p*-values are presented in Supplementary File S1). As shown, there was a positive correlation between MTHF and (MeTHF + THF) in both tumors (Figure 5a) and mucosa (Figure 5b). In tumors, there was no correlation between gene expression and folate levels, whereas in mucosa, there was a significant negative correlation between *TYMS* expression and MTHF levels as well as between *MTHFD1L* expression and both MTHF and (MeTHF + THF) levels. In tumors, a significant negative correlation was seen between *MTHFS* and *ABCC3* as well as between *MTHFS* and *GGH*.

**Figure 6:**
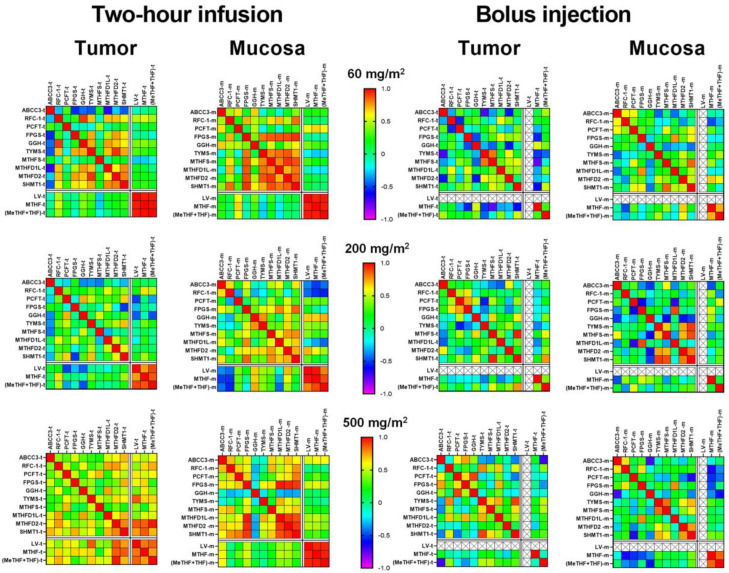
Heat maps showing the correlation between gene expression and folate levels in tumor and mucosa tissues after administration of different dosages of LV given either as two-hour infusion (**left panels**) or bolus injection (**right panels**). The strongest positive correlation (1.0) is shown in red whereas the strongest negative correlation (−1.0) is shown in purple. LV in tissue was not analyzed in the bolus group.

The correlation between gene expression and folates levels was also analyzed in tumor and mucosa of patients subjected to increasing dosage of LV given as two-hour infusion or bolus injection. The results are presented as heat maps in Figure 6 (correlation coefficients and *p*-values are presented in Supplementary File S1). As shown, the correlation patterns differed greatly according to administration regimen. After two-hour infusion of LV, there was a strong, positive correlation between LV, MTHF, and (MeTHF + THF) in both tumors and mucosa at all dosages. After bolus injection of LV, there was no correlation between MTHF and (MeTHF + THF) in tumors at any dosage, however, there was a correlation in the mucosa at dosages 60 and 500 mg/m^2^. 

Compared to baseline values, there were generally more gene expression differences after two-hour infusion than after bolus injection, although the number of correlations increased with increasing LV infusion dosages. The differences consisted almost exclusively of significant positive correlations, which were especially pronounced in tumors after two-hour infusion of 500 mg/m^2^ LV where a strong correlation was seen between *TYMS*, *MTHFD2*, and *SHMT1* and each folate (i.e., LV, MTHF, and MeTHF). In contrast, there were no positive correlations between gene expression and folate levels in tumors after 500 mg/m^2^ bolus injection of LV. Negative correlations were seen between expression of *ABCC3* and *MTHFD1L* (infusion 60 mg/m^2^) and between *ABCC3* and *TYMS* (bolus 60 mg/m^2^) in tumors, as well as in mucosa between *ABCC3* and *GGH* (bolus 500 mg/m^2^). Significant, negative correlations were also seen in tumors between *ABCC3* and MeTHF (bolus 500 mg/m^2^), and between *MTHFS* and MTHF (bolus 60 mg/m^2^). 

### 3.9. Impact of Ischemic Time on Gene Expression

As a restricted blood supply during the surgical procedure will induce ischemia, the time elapsed from vessel stop to biopsy excision may affect gene expression and folate levels in affected tissue. When the ischemic time was analyzed according to LV administration procedure, the results showed that it was shorter in the bolus group than in the two-hour infusion group; the difference being significant at 60 and 500 mg/m^2^ LV (Table 3). 

Because a change has occurred over time in favor of laparoscopic techniques over open procedures in colon resection, the infusion group consisted of more patients undergoing laparoscopic surgery (14/30) than the bolus group (3/27). In the infusion group, laparoscopic surgery resulted in significantly longer ischemic time than open surgery (median time 133 min, range 63–203 versus 75 min, range 27–205, *p* = 0.0044). Among patients subjected to open surgery, the ischemic time was significantly shorter when LV was given as bolus injection compared to two-hour infusion (median time 43 min, range 17–120 versus 75 min, range 27–205, *p* = 0.005). 

To evaluate if the ischemic time had effect on the analyzed genes, the expression levels in adjacent mucosa (obtained 10 cm from the tumor) were compared to those of matched mucosa obtained at the resection margin (assumed not to be subjected to ischemia) of patients subjected to two-hour infusion of LV. No significant differences between the two groups were found, neither when all patients were included (Table 4), nor when the patients were sub-grouped by LV dosage. When the adjacent mucosa/resection margin mucosa gene expression ratio was calculated and correlated to ischemic time, no significant correlation was found for any gene (Table S3). 

### 3.10. Polyglutamated/Monoglutamated Folate Ratio after Two-Hour Infusion versus Bolus Injection of LV

**Figure 7:**
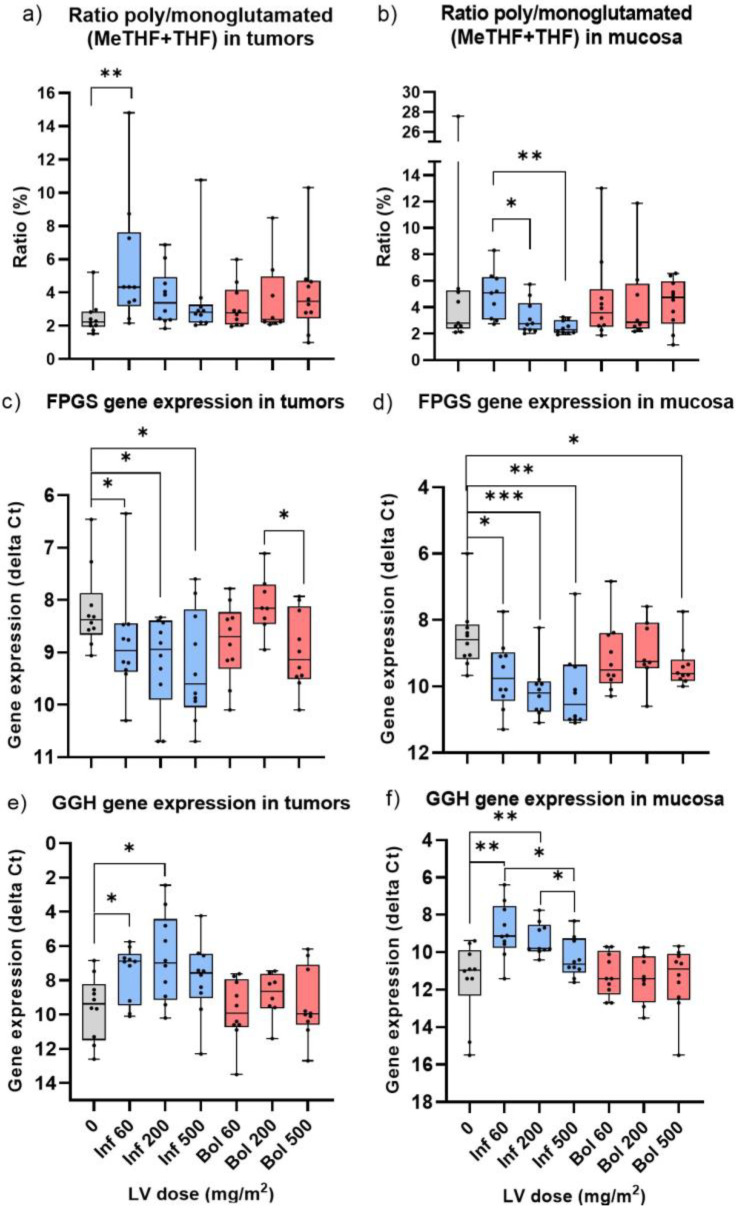
Ratio of polyglutamated/monoglutamated folates, *FPGS* gene expression, and *GGH* gene expression in tumors (**a**,**c**,**e**) and mucosa (**b**,**d**,**f**) of patients receiving no LV, as well as different doses of LV given as two-hour infusion (blue boxes) or bolus injection (red boxes). * *p* ≤ 0.05; ** *p* ≤ 0.01, *** *p* ≤ 0.001.

The total level of polyglutamated folates (i.e., including two or more glutamate chains) was determined by comparing the level of monoglutamated folates in a sample before and after conjugase treatment (which results in degradation of all folate polyglutamates to monoglutamates). The polyglutamated/monoglutamated folate ratio was determined in tumor and mucosa of patients receiving no LV (baseline), as well as different doses of LV given as two-hour infusion or bolus injection (Figure 7). The results showed that in both tumor and mucosa, (MeTHF + THF) were highly polyglutamated (Figure 7a,b) whereas LV had a ratio close to one (i.e., was not polyglutamated). The (MeTHF + THF) polyglutamate pool was higher compared to baseline but decreased with increasing concentration of LV given as two-hour infusion (Figure 7a,b), and the difference between dosage groups was significant in mucosa (Figure 7b). In the bolus group, however, there was no significant difference according to LV dosage and the polyglutamated (MeTHF + THF) level remained relatively high at 500 mg/m^2^. 

The genes *FPGS* and *GGH*, which encode enzymes that add and remove glutamates on folate derivatives, had lower and higher expression, respectively, compared to baseline after two-hour infusion of LV, but not after bolus injection (Figure 7c–f). In tumor samples obtained after two-hour infusion, the expression of *FPGS* was positively correlated with the polyglutamated/monoglutamated (MeTHF + THF) ratio (r = 0.48, *p* = 0.0071). 

## 4. Discussion

The presented study is comparing two study cohorts; one group which received a rapid bolus injection of folate before surgery and the second in which the patients received folates as a two-hour infusion before surgery. In concordance with the previous pharmacokinetic study [10], where LV was given as bolus injection, the present study showed that the folate concentration in plasma increased with increasing dose of LV. Plasma LV AUC and MTHF AUC correlated at higher LV concentrations but not at 60 mg/m^2^ LV. Furthermore, increasing dosage of LV led to increasing LV concentrations in mucosa which were higher compared to tumor tissue, and, in addition, LV levels in mucosa and plasma correlated. Consequently, the “mucosa LV/plasma LV AUC” ratio was higher than the “tumor LV/plasma LV AUC” ratio and increased with dosage. In contrast, the “tissue (MeTHF + THF)/tissue LV” ratio decreased with increasing LV dosage, i.e., the (MeTHF + THF) level was not proportional to the LV dosage. These results indicate that the enzyme systems that convert LV to MeTHF either became saturated or inhibited, which calls into question whether high LV dosages are more beneficial to the patient than low dosages if given as two-hour infusion.

Saturation at high concentrations of LV may be achieved, either due to increased uptake, or due to competition between the unnatural (R) and natural (S) stereodiaisomers. It is known that the R and S forms of racemic LV display significantly different pharmacokinetic behavior [11] and data suggest saturation of the metabolic conversion of S-LV when large doses of LV are administered [12]. R-LV accumulates to a high plasma concentration and might interfere with the transport and metabolism of S-LV [13]. The consequence of the extensive accumulation of the inactive R isomer with high dose of LV is not yet known. However, an earlier study by Schilsky et al. [12] clearly illustrated that intravenous and oral administration of high dose LV resulted in very different profiles of circulating reduced folates. The differences could largely be attributed to the slow plasma clearance of R-LV. A two-hour infusion resulted in prolongation of the plasma half-life for S-LV and the data suggested that conversion of LV to MTHF became saturated following high-dose LV. 

Although most of the R-LV will be excreted after accumulation in plasma, a part may be transported into tissues where it is converted to 10-formylTHF due to chemical reactions (in contrast to enzymatic conversion of S-LV). This subsequently leads to intracellular generation of the natural form of MTHF [14]. Direct uptake of MTHF from plasma also contributes to the intracellular levels of MTHF although this uptake will be inhibited by increasing levels of LV [15]. Inside cells, MTHF is much less effective than LV as a precursor for MeTHF [16]. High concentration of R-LV could thus interfere with the biological activity of S-LV or MTHF. In line with these results, an early study from our group using a rat model showed that treatment with high dose R-LV was associated with a slower tumor growth whereas high dose natural S-LV promoted growth. The increase in tumor growth was associated with a significant increase in TYMS activity [17].

Following administration of racemic LV, the intracellular concentrations of natural folates will depend on folate availability, efficiency of folate influx and efflux, folate polyglutamation, and folate metabolism. These processes are regulated by folate-associated gene expression (Figure 1). As shown in the figure, the folate metabolism is compartmentalized in the mitochondria, the cytosol, and the nucleus [18,19]. Uptake of folates including LV and MTHF occurs via the reduced folate carrier 1 (RFC-1), the proton-coupled folate transporter (PCFT) or through folate receptors (FR1, FR2, and FR3). While RFC-1 is active at physiological pH, PCFT functions optimally at lower pH (5.0–5.5) [20]. The folate receptors transport folates via receptor-mediated endocytosis at neutral to mildly acidic pH. Interestingly, RFC-1 also appears to be capable of transporting monoglutamated folates out of the cells [21]. During folate deficiency, an increased expression of *RFC-1* and *PCFT* transporters has been reported [22]. Under these circumstances, the effect of the RFC-1 efflux mechanism may be detrimental. Folate over-supplementation during a long period of time, on the other hand, decreases transcription of *RFC*, *PCFT* and folate receptors leading to a significant down-regulation in intestinal folate uptake [23]. While RFC-1 and PCFT favor in-transport of S-LV [24,25], human FR1 and FR2 exhibit different specificities for (6S) and (6R) diastereoisomers of folates such as MTHF and LV [20]. The present study showed that *PCFT* gene expression was higher in adjacent mucosa compared to tumors indicating a lower pH in the tumor environment as has been reported [26]. Since influx of LV seems to be pH-dependent [27], the higher levels of LV in mucosa may be linked to the high *PCFT* expression. In tumors, the expression of *PCFT* decreased with increasing infusion dose of LV, indicating a downregulated in-transport of folate through this transporter which was not seen after bolus injection of LV. *RFC-1* expression, on the other hand, did not differ significantly depending on whether LV was given as a bolus or infusion.

The intracellular folate concentrations are regulated by the enzymes FPGS and GGH [28]. FPGS catalyzes the addition of multiple glutamates to tetrahydrofolate derivatives with a preference for MeTHF over LV [29]. Polyglutamation by FPGS increases the cellular retention of MeTHF but also enhances the stabilization of its ternary complex with TYMS and FdUMP [30,31]. In human tissues, *FPGS* encodes two enzymatic isoforms located in the cytosol and mitochondria, respectively [32]. Mitochondrial FPGS is needed because polyglutamated folates in the cytosol cannot be transported across the mitochondrial membrane. The enzyme GGH is located in the lysosomes where it hydrolyzes the γ-glutamyl tail of folate polyglutamates thereby yielding monoglutamated folate forms [33]. The results showed lower *FPGS* and higher *GGH* expression in tumor and mucosa of patients receiving two-hour infusion compared to bolus injection of LV, indicating that infusion of LV resulted in shorter polyglutamation chains of MeTHF than bolus injection. Furthermore, the higher the infusion dosage of LV, the lower the polyglutamate/monoglutamate (MeTHF + THF) ratio. These results are concordant with those of Zhang et al., who reported a shift from longer to shorter polyglutamate chain lengths at higher concentration of LV or longer exposure time [34]. In contrast, polyglutamation of (MeTHF + THF) did not seem to be affected when patients received increasing dosages of bolus LV. Interestingly, a study by Wright et al. [35] using a murine mammary adenocarcinoma model demonstrated that LV administrated as a bolus injection resulted in an expanded pool of MeTHF and towards longer polyglutamate chains suggesting stronger TYMS inhibition. In contrast, continuous infusion of LV provided higher MeTHF AUC than the bolus injection. 

Thus, infusion with high concentrations of LV over a long period of time may result in saturation of the cellular metabolism of S-LV as well as a shift from long-chained to short-chained folate polyglutamates due to decreased expression of *FPGS* and increased expression of *GGH*. This, in turn, may lead to an increased efflux of monoglutamated forms of reduced folate derivatives through transporters like RFC-1 (a high-affinity folate transporter) or membrane-bound efflux pumps, e.g., ABCC3 (a low-affinity folate transporter) [21,36,37]. The results showed that the *ABCC3* expression in general was higher in mucosa than tumors and followed the expression pattern of *PCFT*. The reason for the high expression of these genes in mucosa might relate to a state of hypoxia, which is known to induce overexpression of *ABCC3* as well as acidosis [36,38]. The *ABCC3* expression was higher in tumors after 500 mg/m^2^ LV given as bolus injection compared to two-hour infusion, and a strong, negative correlation was seen between *ABCC3* expression and (MeTHF + THF). This might indicate an overload of folates leading to their extrusion at the highest bolus LV dosage. 

As discussed, an increase of MeTHF in tissue after LV administration is depending on levels of LV and MTHF in plasma, folate uptake and polyglutamation, but also on the intracellular folate metabolism. Field et al. previously demonstrated that S-LV is converted to MeTHF in two steps, catalyzed by the enzymes MTHFS and MTHFD1, respectively [39]. The MTHFS activity may in part account for tissue-specific differences in folate accumulation by affecting folate turnover rates [40]. MTHFS activity has been identified in the cytosol as well as in the mitochondrial matrix, the latter of which is crucial for the entry of LV into the mitochondrial folate pool [41]. Thus, the LV metabolism is dependent on the MTHFS activity but is also affected by the level of 10-formylTHF which strongly inhibits the MTHFS activity [39]. Cell experiments suggest that the ABCC3 protein has a preference for 10-formylTHF compared to other reduced folates [42]. If that holds true, an increased expression of ABCC3 might lower intracellular levels of 10-formylTHF and lead to less inhibition of MTHFS and an increased conversion rate of LV to MeTHF. The results showed no significant difference in *MTHFS* expression between tumors and mucosa at baseline, and no significant differences in tumors by LV dosage, regardless of administration regime. In mucosa samples, however, the *MTHFS* expression increased significantly after two-hour infusion compared to baseline, but gradually decreased by dosage. The decreased expression may have contributed to the high levels of LV found in the mucosa after two-hour infusion.

The recently discovered *MTHFD2* gene encodes a mitochondrial, bifunctional enzyme which catalyzes conversion of MeTHF to 10-formylTHF in two steps [43]. A high cellular *MTHFD2* expression may lead to a high 10-formylTHF level which in turn may inhibit the enzyme MTHFS thereby decreasing the metabolism of LV. The *MTHFD1L* gene encodes another mitochondrial enzyme which generates formate and THF from 10-formylTHF. Elevated expression of both *MTHFD2* and *MTHFD1L* is associated with poor prognosis in CRC [44,45]. The results showed that in concordance with previous results, the *MTHFD2* and *MTHFD1L* expression was higher in tumors compared to mucosa at baseline but there was no significant difference in expression in relation to LV administration regimen or dosage. A large variation in *MTHFD2* expression was noted in both tumor and mucosa which indicates that the gene might be useful as a prognostic marker. 

Serine hydroxymethyltransferases (SHMT) takes part in the cytosolic, mitochondrial, and nuclear folate metabolism by utilizing serine and THF to generate MeTHF and glycine. In the cytosol, SHMT1 seems to be a metabolic switch that gives de novo dTMP synthesis higher metabolic priority than the methyl cycle, when activated [46]. This is accomplished by preferably providing one-carbon units for de novo dTMP synthesis and by competing with the enzyme MTHFR for MeTHF as well as by binding MTHF with high affinity leading to inhibition of the methyl cycle. The results showed higher *TYMS* expression in tumors compared to mucosa at baseline, but no significant difference in tumoral *TYMS* expression after administration of LV. Higher *TYMS* expression was seen in mucosa after two-hour infusion of LV compared to bolus injection. Furthermore, after two-hour infusion of 500 mg/m^2^ LV, a strong positive correlation was seen in tumors between MTHF and (MeTHF + THF) as well as between each folate and *SHMT1*, *MTHFD2*, and *TYMS* expression. No correlation was found between MTHF and (MeTHF + THF) in the bolus group, however. This could indicate activation of the metabolic switch leading to increased dTMP synthesis in the bolus group and a higher probability of TYMS inhibition in response to 5-FU treatment. Inhibition of SHMT1 by high levels of MTHF in combination with low *FPGS* and high *GGH* expression, which were seen in tissues of patients receiving LV as two-hour infusion, would lead to low levels of polyglutamated MeTHF. As reported previously, maximal inhibition of TYMS by the 5-FU metabolite FdUMP in CRC requires excess polyglutamated MeTHF [47]. Administration of LV or [6-R] 5,10-MeTHF (arfolitixorin) [11] as bolus injections during 5-FU-based treatment may be more advantageous than two-hour infusion in this regard.

### Strengths and Limitations of Study

One of the strengths of this prospective study was that collection of tissue and blood samples was performed according to a strict standardized protocol. There was a limited number of patients in the study, however, these patients were carefully monitored by The Swedish Medical Product Agency which approved the study. There is a possibility that variation of folate levels in mucosa and tumor to some extent related to variation in ischemic time. During time, the surgical routine has changed, and most colon cancers are now operated with minimal invasive techniques, in which the vessels are clamped at an early stage of the operation, as a contrast to the open procedures. The mean ischemic time was therefore, as shown, significantly longer in the infusion therapy group, in which 47% of patients had been subjected to laparoscopic surgery, compared to 11% of bolus injection group. However, when gene expression in biopsies collected from the resection margin (with intact circulation until biopsy collection) were compared with adjacent mucosa (affected by ischemia) the results were very similar, hence, we consider the variation of ischemic time of minor importance for the results of the present study. The distribution of patients with right- and left-sided colon cancer differed between the groups, which may have contributed to differences in folate and gene expression levels. 

Due to the limited number of patients, however, it was not possible to perform statistical analyses on patients sub-grouped by tumor location. Although there was a skewness in the proportion of right versus left-sided tumors, which is due entirely to the fact that the patients were collected in a consecutive order without randomization of sidedness, there is no difference in the treatment of patients if they are referred to 5-FU based therapy [2]. LV was not measured in tissue in the bolus injection study; thus, it was only possible to compare tissue levels of MTHF and MeTHF in relation to administration regimens which was a limitation. Another limitation was the lack of data on FR expression. Since FR1 and FR2 exhibit different specificities for (6S) and (6R) diastereoisomers of folates [20], it would be interesting to investigate whether expression of these genes contributes to the differences seen in folate levels between two-hour infusion and bolus injection of racemic LV.

## 5. Conclusions

Identification of predictive markers that can be used for optimal administration of 5-FU modulators such as LV will improve chemotherapy and outcome in colon cancer patients. The aim of study was to compare pharmacokinetics and gene expression in tissue and blood of colon cancer patients subjected to a two-hour infusion or bolus injection of LV. To our knowledge, this is the first study addressing differences related to these LV administration regimens, with special emphasis on folate polyglutamation and associations between folates and gene expression. Although each of the administered administration regimens may have potential benefits, several results point to the benefit of the bolus regimen. For instance, saturation of (MeTHF + THF) levels was seen after two-hour infusion at higher LV dosages, but not after bolus injection. Furthermore, lower levels of polyglutamated (MeTHF + THF) were found in tumors after two-hour infusion compared to bolus injection. The reason for this difference seems to be related to a decreased expression of *FPGS*, and increased expression of *GGH*, in response to increased dosage of LV given as two-hour infusion. While a strong, positive correlation was found between MTHF and (MeTHF + THF) levels as well as between folate levels and expression of *SHMT1*, *MTHFD2*, and *TYMS* after two-hour infusion of 500 mg/m^2^ LV, no such correlation was seen after bolus injection. This might indicate activation of a metabolic switch favoring dTMP synthesis over the methyl cycle after bolus injection. However, the possible impact of these mechanisms when LV is administered in combination with 5-FU needs to be investigated in an extended prospective study.

## Figures and Tables

**Table 1:** Comparison of LV levels in tumor and mucosa of patients treated with two-hour infusion of LV.

LV Dosage (mg/m^2^)	LV Level in Tumor (pmol/g), Median (Range)	LV Level in Mucosa (pmol/g), Median (Range)	*p* ^a^
60	3607 (1514–22310)	4409 (3178–45928)	0.0098
200	9126 (3725–49189)	20326 (9783–73719)	0.064
500	29723 (14978–83321)	96437 (27652–207297)	0.014

^a^ Wilcoxon matched-paired signed rank test.

**Table 2:** Comparison of folate levels in tumor and mucosa of patients treated with two-hour infusion versus bolus injection of LV. Vertical *p*-values are reflecting infusion vs. bolus in each group.

	LV Dosage (mg/m^2^)	Admini-stration	Folate Level in Tumor (pmol/g), Median (Range)	Folate Level in Mucosa (pmol/g), Median (Range)	*p* ^a,b^
MTHF	60	Infusion	2946 (1557–16707)	2371 (1447–14838)	0.77
		Bolus	1274 (935–1560)	1000 (506–2194)	0.43
		*p* ^c,d^	0.0002	0.0013	
(MeTHF + THF)	60	Infusion	2213 (1087–10720)	1393 (599–8736)	0.064
		Bolus	2651 (1001–3493)	1726 (1065–2856)	0.037
		*p* ^c,d^	0.79	0.47	
MTHF	200	Infusion	6422 (2526–22,753)	4971 (3711–30,230)	0.49
		Bolus	2310 (849–3457)	2246 (1404–2822)	0.46
		*p* ^c,d^	0.0016	0.0004	
(MeTHF + THF)	200	Infusion	2597 (1073–11,277)	2262 (1302–5138)	0.56
		Bolus	3620 (2639–11,184	2306 (1515–2928)	0.0078
		*p* ^c,d^	0.35	0.56	
MTHF	500	Infusion	10988 (8342–37,823)	19884 (8136–46,085)	0.16
		Bolus	3392 (2551–7645)	2956 (2195–6026)	0.57
		*p* ^c,d^	0.0002	0.0003	
(MeTHF + THF)	500	Infusion	5047 (2052–9492)	4995 (1503–14,360)	0.56
		Bolus	4909 (2765–7713)	3032 (2241–4670)	0.019
		*p* ^c,d^	0.73	0.096	

^a^ Comparison of tumor and mucosa; ^b^ Wilcoxon matched-paired signed rank test; ^c^ Comparison of infusion and bolus administration; ^d^ Wilcoxon/Mann–Whitney U test.

**Table 3:** Ischemic time by LV administration procedure.

Dose LV (mg/m^2^)	Time (Minutes), Median (Range)					*p* ^a^
	Two-Hour Infusion	n		Bolus Injection	n	
60	120 (60–181)	10		42 (22–120)	10	0.0015
200	84 (27–205)	10		50 (20–175)	7	0.41
500	96 (66–197)	10		45 (17–105)	7	0.0054

^a^ Wilcoxon/Mann–Whitney U test.

**Table 4:** Median gene expression in adjacent mucosa compared to mucosa at resection margin.

Gene	Gene expression (ΔCt), Median (Range)		*p* ^a^
	Mucosa^adj^	Mucosa^res^	
*ABCC3*	6.83 (4.84–11.01)	6.80 (4.73–8.80)	0.82
*RFC-1*	10.87 (9.12–12.96)	10.88 (8.09–12.98)	0.89
*PCFT*	10.92 (7.17–16.32)	11.52 (4.36–16.86)	0.27
*FPGS*	10.12 (7.22–11.26)	10.13 (7.37–11.42)	0.75
*GGH*	9.68 (6.39–11.58)	9.74 (5.96–12.96)	0.45
*TYMS*	10.40 (6.70–11.91)	10.35 (7.68–12.82)	0.63
*MTHFS*	11.75 (9.61–13.26)	11.65 (8.10–13.19)	0.63
*MTHFD1L*	13.56 (10.22–15.23)	13.87 (10.80–15.09)	0.74
*MTHFD2*	10.66 (7.11–13.08)	10.05 (6.16–13.30)	0.47
*SHMT1*	9.26 (6.10–11.01)	9.07 (4.34–10.58)	0.57

^a^ Wilcoxon matched-pairs signed rank, Mucosa^adj^ = adjacent mucosa, Mucosa^res^ = resection margin mucosa.

## Data Availability

The data that support the findings of this study are available on reasonable request from the corresponding author, EBL. The data are not publicly available due to restrictions regarding information that could compromise the privacy of research participants.

## Supplementary Materials

The following supporting information can be downloaded at: https://www.mdpi.com/article/10.3390/cancers15010258/s1, Table S1: Clinical and histopathological characteristics of the study groups; Table S2: List of analyzed genes and assay IDs, Table S3; Correlation between adjacent/resection margin mucosa gene expression ratio and ischemic time, Supplementary File S1: Excel file showing correlation coefficients (r) and *p*-values related to the heat maps in Figure 6 and Figure 7.
