# Supplementary material for: Increasing Dosage of Leucovorin Results in Pharmacokinetic and Gene Expression Differences When Administered as Two-Hour Infusion or Bolus Injection to Patients with Colon Cancer
Source: Cancers (Basel). 2022 Dec 30;15(1):258. doi: 10.3390/cancers15010258 (PMC9818718; doi:10.3390/cancers15010258)
Supplement: Supplementary file 1 [file cancers-15-00258-s001.zip › cancers-2103321-supplementary/cancers-2103321-supplementary/Supplementary File S1.pdf]

	ABCC3-t	ABCC3-m	RFC-1-t	RFC-1-m	PCFT-t	PCFT-m	FPGS-t	FPGS-m	GGH-t	GGH-m	TYMS-t	TYMS-m	MTHFS-t	MTHFS-m	MTHFD1L-t	MTHFD1L-n	MTHFD2-t	MTHFD2 -m	SHMT1-t	SHMT1-m	LV-t	LV-m	MTHF-t	MTHF-m	leTHF+THF	eTHF+THF)
ABCC3-t		0,255927	0,62472	0,935739	0,063802	0,719357	0,014603	0,706386	0,217025	0,280479	0,352009	0,648325	0,292955	0,297951	0,712574	0,722683	0,840529	0,47598	0,260481	0,98818			0,670621	0,111338	0,023078	0,091
ABCC3-m	0,255927		0,172281	0,046668	0,115244	0,757796	0,058831	0,405015	0,310386	0,018034	0,62779	0,755566	0,591894	0,67416	0,073853	0,859407	0,548231	0,45704	0,658382	0,73138			0,979571	0,69062	0,87378	0,922997
RFC-1-t	0,62472	0,172281		0,05576	0,810593	0,639034	0,408072	0,549268	0,962	0,362116	0,006952	0,668698	0,195146	0,444256	0,031055	0,605964	0,726479	0,137398	0,098895	0,055238			0,877058	0,26853	0,225794	0,594465
RFC-1-m	0,935739	**0,046668**	0,05576		0,56503	0,833136	0,285947	0,047757	0,613456	0,642946	0,203249	0,8794	0,354243	0,72068	0,117626	0,556811	0,829382	0,553654	0,302155	0,463177			0,929176	0,060872	0,797319	0,287658
PCFT-t	0,063802	0,115244	0,810593	0,56503		0,214935	0,045458	0,275785	0,000417	0,208774	0,495491	0,542216	0,772161	0,951055	0,270459	0,643589	0,623092	0,676977	0,244949	0,898973			0,705508	0,742883	0,662784	0,549873
PCFT-m	0,719357	0,757796	0,639034	0,833136	0,214935		0,969884	0,660624	0,060638	0,724791	0,375091	0,434793	0,853888	0,380659	0,937005	0,703477	0,649328	0,256257	0,346829	0,039666			0,480679	0,145916	0,866654	0,214629
FPGS-t	**0,014603**	0,058831	0,408072	0,285947	**0,045458**	0,969884		0,154625	0,165099	0,303965	0,630894	0,664842	0,547614	0,435769	0,020299	0,258162	0,734669	0,536168	0,930725	0,712732			0,974872	0,679632	0,308869	0,474357
FPGS-m	0,706386	0,405015	0,549268	**0,047757**	0,275785	0,660624	0,154625		0,201037	0,936201	0,499639	0,482562	0,707424	0,887573	0,097796	0,008272	0,453179	0,953765	0,418598	0,581427			0,210839	0,19124	0,781397	0,979081
GGH-t	0,217025	0,310386	0,962	0,613456	**0,000417**	0,060638	0,165099	0,201037		0,285019	0,673445	0,600198	0,691098	0,842444	0,294782	0,360423	0,686061	0,809932	0,571788	0,952464			0,693531	0,832794	0,705284	0,566856
GGH-m	0,280479	**0,018034**	0,362116	0,642946	0,208774	0,724791	0,303965	0,936201	0,285019		0,582585	0,752864	0,148924	0,379039	0,207309	0,612649	0,294381	0,770885	0,814687	0,267791			0,903114	0,322953	0,679466	0,077391
TYMS-t	0,352009	0,62779	**0,006952**	0,203249	0,495491	0,375091	0,630894	0,499639	0,673445	0,582585		0,605549	0,042314	0,450673	0,018362	0,604611	0,593153	0,110793	0,000417	0,042717			0,637941	0,614456	0,156023	0,810037
TYMS-m	0,648325	0,755566	0,668698	0,8794	0,542216	0,434793	0,664842	0,482562	0,600198	0,752864	0,605549		0,279052	0,002771	0,748652	0,667685	0,845801	0,064849	0,485103	0,022903			0,839346	0,415853	0,109495	0,707287
MTHFS-t	0,292955	0,591894	0,195146	0,354243	0,772161	0,853888	0,547614	0,707424	0,691098	0,148924	**0,042314**	0,279052		0,150113	0,301439	0,892819	0,581084	0,160717	0,046916	0,106728			0,576404	0,987345	0,15337	0,770272
MTHFS-m	0,297951	0,67416	0,444256	0,72068	0,951055	0,380659	0,435769	0,887573	0,842444	0,379039	0,450673	**0,002771**	0,150113		0,691064	0,766852	0,629569	0,247282	0,627013	0,055615			0,750595	0,811075	0,013821	0,975916
MTHFD1L-t	0,712574	0,073853	**0,031055**	0,117626	0,270459	0,937005	**0,020299**	0,097796	0,294782	0,207309	**0,018362**	0,748652	0,301439	0,691064		0,208366	0,361139	0,81436	0,165413	0,210141			0,964055	0,68656	0,440208	0,913128
MTHFD1L-m	0,722683	0,859407	0,605964	0,556811	0,643589	0,703477	0,258162	**0,008272**	0,360423	0,612649	0,604611	0,667685	0,892819	0,766852	0,208366		0,239296	0,72723	0,332209	0,994442			0,774516	0,75255	0,671399	0,818668
MTHFD2-t	0,840529	0,548231	0,726479	0,829382	0,623092	0,649328	0,734669	0,453179	0,686061	0,294381	0,593153	0,845801	0,581084	0,629569	0,361139	0,239296		0,117052	0,840656	0,990919			0,468729	0,610279	0,511526	0,288509
MTHFD2 -m	0,47598	0,45704	0,137398	0,553654	0,676977	0,256257	0,536168	0,953765	0,809932	0,770885	0,110793	0,064849	0,160717	0,247282	0,81436	0,72723	0,117052		0,03471	0,012329			0,332619	0,892935	0,367578	0,799712
SHMT1-t	0,260481	0,658382	0,098895	0,302155	0,244949	0,346829	0,930725	0,418598	0,571788	0,814687	**0,000417**	0,485103	**0,046916**	0,627013	0,165413	0,332209	0,840656	**0,03471**		0,069687			0,55746	0,744386	0,354685	0,782479
SHMT1-m	0,98818	0,73138	0,055238	0,463177	0,898973	**0,039666**	0,712732	0,581427	0,952464	0,267791	**0,042717**	**0,022903**	0,106728	0,055615	0,210141	0,994442	0,990919	**0,012329**	0,069687				0,678861	0,32211	0,211109	0,330751
LV-t																										
LV-m																										
MTHF-t	0,670621	0,979571	0,877058	0,929176	0,705508	0,480679	0,974872	0,210839	0,693531	0,903114	0,637941	0,839346	0,576404	0,750595	0,964055	0,774516	0,468729	0,332619	0,55746	0,678861				0,332132	0,791423	0,787659
MTHF-m	0,111338	0,69062	0,26853	0,060872	0,742883	0,145916	0,679632	0,19124	0,832794	0,322953	0,614456	0,415853	0,987345	0,811075	0,68656	0,75255	0,610279	0,892935	0,744386	0,32211			0,332132		0,622353	0,003992
(MeTHF+THF)-t	**0,023078**	0,87378	0,225794	0,797319	0,662784	0,866654	0,308869	0,781397	0,705284	0,679466	0,156023	0,109495	0,15337	**0,013821**	0,440208	0,671399	0,511526	0,367578	0,354685	0,211109			0,791423	0,622353		0,642415
(MeTHF+THF)-m	0,091	0,922997	0,594465	0,287658	0,549873	0,214629	0,474357	0,979081	0,566856	0,077391	0,810037	0,707287	0,770272	0,975916	0,913128	0,818668	0,288509	0,799712	0,782479	0,330751			0,787659	**0,003992**	0,642415	
