# Supplementary material for: Increasing Dosage of Leucovorin Results in Pharmacokinetic and Gene Expression Differences When Administered as Two-Hour Infusion or Bolus Injection to Patients with Colon Cancer
Source: Cancers (Basel). 2022 Dec 30;15(1):258. doi: 10.3390/cancers15010258 (PMC9818718; doi:10.3390/cancers15010258)
Supplement: Supplementary file 1 [file cancers-15-00258-s001.zip › cancers-2103321-supplementary/cancers-2103321-supplementary/Supplementary/Supplementary Materials.pdf]

Supplementary Materials: The following supporting information can be downloaded at: www.mdpi.com/xxx/s1, Table S1: Clinical and histopathological characteristics of the study groups; Table S2: List of analyzed genes and assay IDs, Table S3; Correlation between adjacent/resection margin mucosa gene expression ratio and ischemic time, Supplementary File S1: Excel file showing correlation coefficients (r) and p-values related to the heat maps in Fig. 6 and Fig. 7.

Table S1. Clinical and histopathological characteristics of the study groups

Parameter	LV administration regime		p	No LV given (n = 10)	Total (n = 68)
	Two-hour infusion (n = 30)	Bolus injection (n = 28)			
Age, median (range)	68 (50-89)	68 (43-89)	0.80[b]	73 (59-88)	70 (37-89)
Gender, n (%)					
Female	11 (37)	15 (54)		4 (40)	30 (44)
Male	19 (63)	13 (46)	0.20[c]	6 (60)	38 (56)
Tumor location, n (%)[a]					
Right side	8 (27)	18 (64)		7 (70)	32 (47)
Left side	22 (73)	10 (36)	0.0016[c]	3 (30)	35 (51)
Disease stage, n (%)[a]					
I	3 (10)	2 (7)		0	5 (7)
II	9 (30)	14 (50)		3 (30)	26 (38)
III	18 (60)	10 (36)		7 (70)	35 (51)
IV	0	2 (7)	0.14[c]	0	2 (3)

[a]Percentage does not add up due to rounding; [b]Wilcoxon/Kruskal-Wallis tests; [c]Pearson test

Table S2. List of analyzed genes and assay IDs.

Gene category	Gene	Gene name	Assay ID
Folate transport	ABCC3/MRP3	ATP-binding cassette, subfamily C (CFTR/MRP), member 3	Hs00358656_ml
	SLC19A1/RFC-1	Solute carrier family 19 (folate transporter) member 1; reduced folate carrier 1	Hs00953344_ml
	SLC46A1/PCFT	Solute carrier family 46 (folate transporter), member 1; proton coupled folate transporter	Hs00611081_m1
Folate metabolism	MTHFD1L	Methylenetetrahydrofolate dehydrogenase (NADP+ dependent) 1-like	Hs00383616_ml
	MTHFD2	Methylenetetrahydrofolate dehydrogenase (NAPD+ dependent) 2	Hs00741165_m1
	MTHFS	5,10-Methenyltetrahydrofolate synthetase	Hs00197574_ml
	SHMT1	Serine hydroxymethyltransferase 1	Hs00541038_ml
	TYMS	Thymidylate synthase	Hs00426586_m1
Folate polyglutamation	FPGS	Folylpolyglutamate synthase	Hs00191956_ml
	GGH	Gamma-glutamyl hydrolase (conjugase, folylpolygammaglutamyl hydrolase)	Hs00914163_ml
Reference genes	ACTB	Beta-actin	Hs99999903_ml
	GAPDH	Glyceraldehyde 3-phosphate dehydrogenase	Hs99999905_ml

Table S3. Correlation between adjacent/resection margin mucosa gene expression ratio and ischemic time.

$Mucosa^{adj}/Mucosa^{res}$ gene expression ratio	r^a	P^b
ABCC3	0.12	0.53
RFC-1	0.20	0.29
PCFT	0.30	0.11
FPGS	0.28	0.15
GGH	0.096	0.62
TYMS	0.11	0.58
MTHFS	0.37	0.050
MTHFD1L	0.21	0.27
MTHFD2	0.25	0.18
SHMT1	0.25	0.19

[a]Correlation coefficient, [b]Pearson pairwise correlation, $Mucosa^{adj}$ = adjacent mucosa, $Mucosa^{res}$ = resection margin mucosa

Supplementary File S1

Excel file showing correlation coefficients (r) and p-values related to the heat maps in Fig. 6 and Fig. 7. R- and p-values for each group of patients, i.e., for those not exposed to LV (0), or for those who received 60, 200 or 500 mg/m^2 LV as a two-hour infusion (Inf) or bolus injection (Bol) can be found under each respective tab. Significant p-values and corresponding r-values are marked in red.
